# Weight changes among antiretroviral therapy-naïve people living with human immunodeficiency virus in Lagos, Nigeria

**DOI:** 10.3389/fpubh.2025.1545676

**Published:** 2025-05-13

**Authors:** Oluwatosin Olaseni Odubela, Nasheeta Peer, Nkiruka Nnonyelum Odunukwe, Adesola Zaidat Musa, Babatunde Lawal Salako, Andre Pascal Kengne

**Affiliations:** ^1^Faculty of Health Sciences, Department of Medicine, University of Cape Town, Cape Town, South Africa; ^2^Department of Clinical Sciences, Nigerian Institute of Medical Research, Lagos, Nigeria; ^3^Non-Communicable Diseases Research Unit, South African Medical Research Council, Cape Town, South Africa; ^4^Department of Medicine, University College Hospital, Ibadan, Nigeria

**Keywords:** weight changes, people living with HIV, antiretroviral therapy, naïve, Lagos, Nigeria

## Abstract

**Introduction:**

The advent of antiretroviral therapy (ART) has converted HIV from a death sentence to a chronic disease. Subsequently, weight changes, including the development of overweight/obesity have been observed following ART initiation. Our study aimed to assess weight changes and the associated factors among ART-naïve people living with HIV (PLWH) following enrollment in an ART clinic in Lagos, Nigeria.

**Methodology:**

Data were collected among adult ART-naïve PLWH enrolled at a large ART clinic over 10 consecutive years. Weight changes within the first 6 months of enrolment were determined by actual and relative weight differences expressed in kilogram (kg) and percentages (%) respectively. Weight changes were classified as neutral weight change, weight gain and weight loss. Logistic regressions were applied to identify variables associated with weight changes with statistical significance set at *p* < 0.05.

**Results:**

A total of 6,737 study participants had their weights available at both visits. Most study participants were females (67.2%), employed (83.3%), married (57.1%), and had normal range body mass index (53.5%). Almost half (49.5%) of the study participants gained weight, while 25.5% recorded weight loss. Baseline variables, including viral load ≥ 100,000 copies/ml, CD4 counts ≤ 200 cells/μL, WHO clinical stages 3 and 4, male gender, presence of anaemia and tuberculosis were associated with weight gain after ART initiation.

**Conclusion:**

Considering the high proportion of participants that gained weight, this study highlights the importance of monitoring weight changes following ART initiation. This will facilitate the identification of PLWH at greater risk for cardiometabolic diseases and other weight-related health outcomes.

## Introduction

Globally, approximately 39.9 million people were living with HIV (PLWH) in 2023, with a significant proportion on effective antiretroviral therapy (ART) (1). The advent of ART has transformed HIV/AIDS from a fatal illness to a chronic condition with PLWH having a similar lifespan to the HIV-uninfected population (2). Nevertheless, the survival of PLWH is being undermined by the rising incidence of non-communicable diseases (NCDs). This could reverse some of the anticipated gains of HIV care if not curtailed. The prevalent NCDs in this population include cardiometabolic disorders such as obesity, mental health disorders, chronic pulmonary diseases and chronic kidney diseases among others (3, 4). The drivers of NCDs have been attributed to the effect of antiretrovirals (ARV), lifestyle and behavioral changes (5).

Weight gain, an indicator of successful ARV treatment, has now come under scrutiny. The drawbacks of weight gain include increased risks of insulin resistance and dyslipidaemia (6). Weight gain could also impact mental health, arising from body image issues, anxiety, and low self-esteem (7). Excessive weight gain results in obesity, an independent risk factor for morbidity and mortality related to cardiovascular and other diseases (6, 8). Newer ARV regimens, especially integrase strand inhibitors (INSTIs), have been associated with significant weight gains among PLWH (9). Furthermore, the adoption of unwholesome lifestyles by PLWH, such as the consumption of unhealthy diets, decreased physical activity, and sedentary work routines is likely to lead to weight gain (10).

In sub-Saharan Africa (SSA), where the HIV burden is highest globally, studies have evaluated weight changes among PLWH following enrollment into care (9, 11). Most studies have aligned with weight gain following ART initiation globally and SSA, with the highest weight gain observed in the first year of ART initiation (6, 12). Attributable factors for the observed weight gain include female gender, black race, clinical status at ART initiation (low CD4 counts, high viral loads, advanced clinical staging of HIV disease), diets, and older age (13–15). However, there is limited evidence on weight changes among PLWH in Nigeria, one of the countries with a high HIV burden on the continent (16, 17). Lagos State, the economic capital of Nigeria, is home to over 18 million residents and an HIV prevalence of 1.3% (18). Our study aimed to assess weight changes and their associated factors following ART initiation among ART-naïve PLWH at an ART clinic in Lagos, Nigeria.

## Materials and methods

### Study setting

The Clinical Sciences Department, within the Nigerian Institute of Medical Research (NIMR) operates an ART clinic. The ART clinic has been in existence since 2002 and cumulatively enrolled more than 25,000 PLWH. The clinic provides care and management to adults, pregnant women, adolescents, and infants exposed or infected with HIV. The ART clinic is located in the country’s economic capital, Lagos State. Services received in the facility are free of charge and adhere to the country’s national guidelines for HIV prevention, treatment, and care.

Data of PLWH accessing care and treatment at the facility were captured in an electronic database and maintained on a server onsite. Each person is assigned a unique identification number, which is used to collate data relating to clinical, therapeutic, and laboratory parameters. At enrolment, patients are offered counseling services (pre- and post-confirmation of HIV diagnosis) and an informed consent process. A consent form is provided to document their choice of the use of their data and biological samples for research purposes. Their responses do not influence the care and treatment received in the facility. The clinic offers only outpatient services while patients requiring admission are referred to facilities closest to their residence or choice.

### Study design, population, and data collection

This is a longitudinal review of data collected among adult ART-naïve PLWH enrolled in the clinic over 10 years (January 1, 2010 – December 31, 2019). Participants were included in the study if they had their weights recorded at baseline and scheduled their first follow-up clinic visit (6 (± 2) months post-enrolment) irrespective of their ART status. Those who were younger than 18 years of age, ART-experienced, or receiving care for post-exposure prophylaxis (PEP) were excluded from the study. All eligible patients attending the clinic were included in the study.

At enrolment and follow-up visits, the nurse took the patients’ vital signs (temperature, weight, height, blood pressure). Weights to the nearest 0.1 kg were measured with patients standing on the weighing scale, barefooted in an erect posture. Heights were recorded with the marker placed at the crown of the head and measurements were taken to the nearest 0.1 cm. Blood pressure (BP) measurements were obtained with the patient in a sitting position (at least 5 min before the assessment) using a digital BP monitor with an appropriate cuff size. The BP readings were taken three times with the average recorded as the patient’s readings while the weights and heights of study participants were observed once.

At baseline and subsequent clinic visits, the clinician conducts a detailed clinical history and physical examination for the study participants. At the baseline visit, the patient’s WHO clinical staging was determined and baseline investigations were conducted (full blood count, cluster of differentiation 4 (CD4) count, viral load, serum creatinine, random blood glucose, Hepatitis B and C screening). Chest X-ray and sputum evaluation were done based on the national guidelines for possible tuberculosis co-infection (19).

Data collected for the study at the baseline clinic visit included sociodemographic characteristics (age, gender, education and marital status, occupation, and year of enrolment), anthropometry (weight, height), blood pressure, comorbidities at presentation (hypertension, diabetes mellitus, tuberculosis, anaemia, Hepatitis B and C), HIV specific characteristics (viral load, CD4 counts, and WHO clinical staging), and laboratory investigations (haemoglobin, Hepatitis B and C) at enrolment in the clinic. Data relating to anthropometric measures (weight and height), HIV-specific characteristics (viral load, CD4 counts and ART status), and anaemia were collected for review at the scheduled follow-up visit.

### Definitions

The study outcome of interest is weight change observed among ART-naïve PLWH at enrolment and 6 months post-ART initiation. Weight changes were assessed by actual and relative weight differences expressed in kilograms (kg) and percentages (%) respectively. Weight changes were classified into 3 categories using weight differences in kilograms and percentages. The categories for weight changes in kg are neutral weight change ±1 kg, weight gain ≥ 1 kg, and weight loss ≥ 1 kg. In terms of weight changes expressed in percentages, the categories were neutral weight change (<5%), weight gain ≥ 5%, and weight loss ≥ 5%. These cut-points were selected following recent studies within the HIV cohort. A study among veterans in the United States of America (USA) recorded a median weight change of 2.7 kg (5 pounds) over a one-year follow-up period post-ART initiation (20). Other studies using weight difference expressed in percentages utilized 5% weight change while another correlated 2.5% weight differentials with mortality at 1 month (21, 22). Due to the short duration of the follow-up (6 months), we aligned our definition of weight changes to prior studies with similar profiles 2.7 kg weight gain within a year of ART initiation or percentage (2.5% or 5%) change. In addition, PLWH with weight loss exceeding 5 and 10% have been classified to be either in WHO Clinical Stage 3 or 4, respectively (23).

Body mass index (BMI) was calculated as the weight (in kilogram, kg) divided by height squared (in metres) – kg/m^2^ (24). BMI was classified as underweight (< 18.5), normal (18.5–24.9), overweight (25.0–29.9), and obese (≥ 30.0) (24).

Hypertension was defined as systolic and/or diastolic BP (SBP and DBP) greater than or equal to 140 mmHg and 90 mmHg, respectively, in addition to prior history of hypertension diagnosis or use of anti-hypertensive medications (irrespective of current BP readings) (25). Anaemia was defined as haemoglobin level less than 10 g/dL (26). Study participants were diagnosed with diabetes mellitus in the presence of a prior diagnosis (from clinical history or medical records) and/or were currently on treatment. Tuberculosis was diagnosed following clinical history, physical examination, chest X-ray findings and sputum examination based on national guidelines (27).

Viral load was classified into two categories (≤ 100,000 and ≥ 100,001 copies per millimetre) while CD4 counts were classified according to the CDC Staging (23).

Study participants were initiated on ART based on the prevailing national treatment guidelines for the care and management of HIV at the time of enrolment into the clinic which have seen numerous revisions throughout the study period (28, 29).

### Statistical analysis

Data were presented in counts and percentages for categorical variables with mean (and standard deviation) or median (and 25th – 75th percentiles) for continuous variables. The data was evaluated for normality and multicollinearity, while Chi-square test was used to determine the relationship between categorical variables and observed weight changes. Student t-test was used to compare the means of variables. We explored the predictors of weight changes among participants who initiated ART using both univariate and multivariate regression analysis (including variables such as age, sex, occupation, marital status, anaemia, ART status, ART regimen, year of enrolment, as well as HIV-specific factors and other clinical variables). The multivariable regression analysis was adjusted for age and gender for the two models (absolute and differential weight changes). Statistical significance was set at *p*-value < 0.05 and 95% Confidence intervals provided. Data was analyzed using Statistical Package for Social Sciences (SPSS) version 29 (IBM SPSS Inc., Chicago, IL).

### Ethical approval

Ethical approval was sought and obtained from the Institutional Review Board (IRB) of the Nigerian Institute of Medical Research (NIMR) [IRB-21-066]. Additionally, ethical approval was also been obtained from the Health Research Committee of the University of Cape Town, South Africa [HREC 176/2022].

## Results

Among 10,516 ART-naïve PLWH enrolled during the study period, 6,737 study participants had their weights available at both baseline and 6-month follow-up clinic visits. The mean age (± SD) of study participants at enrollment was 36.5 (± 9.2) years, with males found to be older than females (40.3 vs. 34.7 years, respectively, *p* < 0.0001). At enrolment, all sociodemographic, laboratory, and HIV-specific factors were significantly different by gender except for educational status. A majority of study participants were females (67.2%), employed (83.3%), married (57.1%), and had normal BMI (53.5%). In addition, hypertension (19.7%) was the most common comorbidity recorded at the baseline visit. Concerning HIV-specific characteristics, 67.7 and 57.4% of participants had viral load ≤100,000 copies/ml and CD4 counts ≥ 201 cells/μL, respectively. A majority of the study participants were either in WHO Clinical Stages 1 or 2 (57.1%) (Table 1).

**Table 1:** Baseline characteristics of study participants at enrollment.

Variables	Total, *n* = 6,737 (%)	Female, *n* = 4,525 (%)	Male, *n* = 2,212 (%)	*p*-value
^*^Mean age (± SD), in years	36.5 (± 9.2)	34.7 (± 8.5)	40.3 (± 9.3)	< 0.001
Age groups (years)				< 0.001
18–24	379 (5.6)	305 (6.7)	74 (3.4)	
25–49	5,800 (86.1)	3,952 (87.3)	1848 (83.5)	
≥ 50	558 (8.3)	268 (6.0)	290 (13.1)	
Educational status				0.279
None/Primary	1,411 (21.1)	971 (21.6)	440 (20.0)	
Secondary	2,918 (43.6)	1951 (43.4)	967 (43.9)	
Tertiary	2,369 (35.4)	1,572 (35.0)	797 (36.1)	
Occupational status				< 0.001
Unemployed/retired	1,118 (16.7)	921 (20.4)	197 (8.9)	
Employed	5,594 (83.3)	3,589 (79.6)	2005 (91.1)	
Marital status				< 0.001
Single	1906 (28.3)	1,268 (28.0)	638 (28.8)	
Married	3,847 (57.1)	2,466 (54.5)	1,380 (62.4)	
Separated	399 (5.9)	308 (6.8)	91 (4.1)	
Widowed	586 (8.7)	483 (10.7)	103 (4.7)	
^#^Median Body mass index (BMI) (25–75 percentile), kg/m^2^	22.0 (19.0–26.0)	23.0 (19.0–26.0)	22.0 (19.0–25.0)	
Body mass index, BMI (kg/m^2^)				< 0.001
Underweight (< 18.5)	589 (10.3)	417 (10.8)	172 (9.1)	
Normal (18.5–24.5)	3,068 (53.5)	1938 (50.4)	1,130 (59.9)	
Overweight (25.0–29.9)	1,418 (24.7)	968 (25.2)	450 (23.8)	
Obese (≥ 30.0)	661 (11.5)	525 (13.6)	136 (7.2)	
Comorbidities present				
Hypertension	1,327 (19.7)	759 (16.8)	568 (25.7)	< 0.001
Diabetes mellitus	83 (1.3)	45 (1.0)	38 (1.8)	0.008
Tuberculosis	339 (5.0)	177 (3.9)	162 (7.3)	< 0.001
Anaemia	2018 (30.0)	1,577 (34.9)	441 (20.0)	< 0.001
Hepatitis B	330 (5.7)	168 (4.4)	162 (8.5)	< 0.001
Hepatitis C	140 (2.5)	100 (2.6)	40 (2.1)	0.039
WHO Clinical Staging				< 0.001
Stage 1	1,105 (19.8)	824 (22.3)	281 (14.8)	
Stage 2	2085 (37.3)	1,413 (38.3)	672 (35.4)	
Stage 3	1914 (34.3)	1,174 (31.8)	740 (39.0)	
Stage 4	482 (8.6)	279 (7.6)	203 (10.7)	
^#^Median CD4 counts (25–75 percentile)	239 (112–394)	255 (126–424)	207 (87–349)	
CD4 counts (cells/μL)				< 0.001
≤ 200	2,866 (42.6)	1793 (39.7)	1,073 (48.6)	
≥ 201	3,858 (57.4)	2,725 (60.3)	1,133 (51.4)	
^#^Median Viral load (25–75 percentile)	22,280 (200–169,947)	17,855 (200–137,588)	37,982 (178–243,930)	
Viral load (copies/ml)				< 0.001
≤ 100,000	4,193 (67.7)	2,971 (71.0)	1,222 (60.8)	
≥ 100,001	2000 (32.3)	1,213 (29.0)	787 (39.2)	
Year of enrollment				0.001
2010	1,366 (20.3)	946 (20.9)	420 (19.0)	
2011	1,319 (19.6)	903 (20.0)	416 (18.8)	
2012	994 (14.8)	671 (14.8)	323 (14.6)	
2013	1,361 (20.2)	937 (20.7)	424 (19.2)	
2014	547 (8.1)	351 (7.8)	196 (8.9)	
2015	472 (7.0)	306 (6.8)	166 (7.5)	
2016	266 (3.9)	172 (3.8)	94 (4.2)	
2017	162 (2.4)	88 (1.9)	74 (3.3)	
2018	159 (2.4)	91 (2.0)	69 (3.1)	
2019	91 (1.4)	60 (1.3)	31 (1.4)	

WHO, World Health Organization; CD4, Cluster of Differentiation 4, SD, standard deviation; ^*^Mean (SD).

^#^Median (25–75 percentile).

The dataset was assessed for normality using the Kolmogorov–Smirnov test, and all variables were normally distributed (*p* < 0.001), the study variables were also evaluated for interactions/collinearity with the collinearity tolerance and statistics variance inflation factor (VIF) values showing low levels for multicollinearity (Supplementary Table 1).

The mean weights (± SD) of study participants at baseline and 6-month clinic visits were 64.7 (±14.4)kg and 66.4 (±14.3) kg, respectively, with the difference found to be statistically significant, *p* < 0.001 (Table 2). The mean BMI (± SD) at baseline and 6 months were 23.6 kg/m^2^ (± 5.8) and 24.9 kg/m^2^ (± 5.1), respectively. The paired sample difference (± SD) of the mean BMIs was −1.2 kg/m^2^ (± 5.1) with the difference found to be significant (p < 0.001). In addition, the proportion of study participants with underweight and normal BMI (BMI < 25.0 kg/m^2^) decreased from 63.8 to 58.2% with about 50% reduction in the prevalence of underweight PLWH at 6 months when compared to the baseline, Table 2. The median weight change among study participants with recorded weight gain was 2.0 (25 – 75th percentile, −1.0 – 5.0) kg. Furthermore, the use of antiretroviral agents was shown to be associated with weight gain and loss irrespective of the regimens initiated (*p* < 0.05), Supplementary Table 2.

**Table 2:** Baseline and 6-month weights and Body mass index (BMI) of study participants.

Variables	Baseline visit	6-month visit
Gender	Mean weight (± SD)	Mean weight (± SD)
Female (4525)	62.7 (±14.4)	64.2 (±14.2)
Male (2212)	68.8 (±13.5)	71.1 (±13.4)
Total (6737)	64.7 (±14.4)	66.4 (±14.3)
Body mass index (BMI), kg/m^2^	N (%)	N (%)
Underweight (≤ 18.5)	589 (10.3)	343 (5.9)
Normal (18.5–24.9)	3,068 (53.5)	3,057 (52.3)
Overweight (25.0–29.9)	1,418 (24.7)	1,650 (28.2)
Obese (≥ 30.0)	661 (11.5)	794 (13.6)
Categorical BMI, kg/m^2^	N (%)	N (%)
Underweight/Normal (< 25.0)	3,657 (63.8)	3,400 (58.2)
Overweight/Obese (≥ 25.0)	2079 (36.2)	2,444 (41.8)

Male study participants had relatively higher weights at both baseline and 6-month visits when compared to their female counterparts (*p* < 0.001). Overall, a significant proportion of participants gained weight (49.5%), while 25.5% lost weight and 25.0% had neutral weight change. This trend was similar for females (weight gain, 47.4%; weight loss, 28.0%; and no change, 24.6%) and males (weight gain, 53.2%; weight loss, 20.4%; and no change, 26.4%). Eight hundred and ninety-three (893) PLWH were yet to commence ART at their 6-month clinic visit, with 38.5% (n = 344) and 35.3% (n = 315) of them recording actual weight gain and loss, respectively (Table 3). Using weight changes by percentage cutoffs, the majority of PLWH had neutral weight change (3 316, 49.2%), followed by those with weight gain (2 390, 35.5%) and weight loss (1 031, 15.3%) (Table 4).

**Table 3:** Participants characteristics presented by actual absolute weight change at 6 months.

Variables	Neutral weight change *N* = 1,696 (%)	Weight gain *N* = 3,322 (%)	Weight loss *N* = 1719 (%)	*p*-value
Gender				< 0.001
Female	1,111 (65.5)	2,146 (64.6)	1,268 (73.8)	
Male	585 (34.5)	1,176 (35.4)	451 (26.2)	
Age group (years)				0.017
18–24	102 (6.0)	168 (5.1)	109 (6.3)	
25–49	1,466 (86.4)	2,846 (85.7)	1,488 (86.6)	
≥ 50	128 (7.6)	308 (9.3)	122 (7.1)	
Education				0.012
None/Primary	315 (18.7)	743 (22.5)	353 (20.7)	
Secondary	731 (43.3)	1,432 (43.4)	755 (44.2)	
Tertiary	641 (38.0)	1,128 (34.1)	600 (35.1)	
Occupation				0.055
Unemployed/Retired	275 (16.3)	526 (15.9)	317 (18.5)	
Employed	1,414 (83.7)	2,784 (84.1)	1,396 (81.5)	
Marital status				< 0.001
Single	505 (29.8)	986 (29.7)	415 (24.1)	
Married	971 (57.3)	1794 (54.0)	1,081 (62.9)	
Separated	85 (5.0)	224 (6.7)	90 (5.2)	
Widowed	135 (8.0)	318 (9.6)	133 (7.7)	
Year of enrollment				0.002
2010	327 (19.3)	702 (21.1)	337 (19.6)	
2011	321 (18.9)	679 (20.4)	319 (18.6)	
2012	238 (14.0)	461 (13.9)	295 (17.2)	
2013	352 (20.8)	646 (19.4)	363 (21.1)	
2014	129 (7.6)	292 (8.8)	126 (7.3)	
2015	130 (7.7)	227 (6.8)	115 (6.7)	
2016	94 (5.5)	107 (3.2)	65 (3.8)	
2017	36 (2.1)	84 (2.5)	42 (2.4)	
2018	46 (2.7)	81 (2.4)	32 (1.9)	
2019	23 (1.4)	43 (1.3)	25 (1.5)	
Co-morbidities present				
Anaemia	426 (25.2)	1,106 (33.4)	486 (28.3)	< 0.001
Tuberculosis	50 (3.0)	241 (7.3)	48 (2.8)	< 0.001
Hypertension	351 (20.7)	608 (18.3)	368 (21.4)	0.016
Diabetes	20 (1.2)	38 (1.2)	25 (1.5)	0.632
Hepatitis B	69 (4.7)	172 (6.1)	89 (6.1)	0.166
Hepatitis C	32 (2.3)	75 (2.7)	33 (2.3)	0.609
WHO staging, at enrollment				< 0.001
Stage 1 and 2	894 (64.9)	1,463 (51.3)	833 (61.4)	
Stage 3 and 4	483 (35.1)	1,390 (48.7)	523 (38.6)	
CD4 counts, cells/μL				< 0.001
≤ 200	607 (35.9)	1,644 (49.6)	615 (35.8)	
≥ 201	1,084 (64.1)	1,672 (50.4)	1,102 (64.2)	
Viral load, copies/ml				< 0.001
≤ 100,000	1,074 (69.3)	1953 (63.6)	1,166 (74.3)	
≥ 100,001	476 (30.7)	1,120 (36.4)	404 (25.7)	
On ART regimen (at 6 month visit)				< 0.001
No	244 (14.4)	334 (10.1)	315 (18.3)	
Yes	1,452 (85.6)	2,988 (89.9)	1,404 (81.7)	

WHO, World Health Organization; CD4, Cluster of Differentiation 4; ART, Antiretroviral therapy, Neutral weight change – (± 1) kg, Weight gain – > (+1) kg, Weight loss - > (−1) kg. The participant’s characteristics are at baseline clinic visit.

Univariable and multivariable logistic regressions were employed to determine the predictors of actual and differential weight gain observed at the 6-month clinic visit. On multivariable regression, the models were adjusted for age and gender (Tables 5 and 6). Study participants who were males (AOR: 1.35, 95% CI: 1.16–1.56), had tuberculosis (AOR: 2.17, 95% CI: 1.56–3.02), anaemia (AOR: 1.21, 95% CI: 1.04–1.40), CD4 counts ≤ 200 cells/μL (AOR: 1.34, 95% CI: 1.17–1.54), viral load ≥ 100,001 copies/ml (AOR: 1.21, 95% CI: 1.04–1.40), and WHO Clinical stages 3 and 4 (AOR: 1.33, 95% CI: 1.15–1.53) at presentation to the ART clinic were associated with actual weight gain at their 6-month follow-up clinic visit. Study participants with co-morbid hypertension (AOR: 0.81, 95% CI: 0.70–0.94) were less likely to have weight gain at their subsequent clinic visit when compared to their baseline weights (Table 5). In addition, PLWH commenced on Nevirapine-based regimen had comparatively higher weight gains than other drug regimens (Table 5).

**Table 4:** Participants characteristics presented by cut point of 5% relative change in weight at 6 months.

Differential weights expressed in percentages, *N* (%)				
Variables	Neutral Weight change *N* = 3,316 (%)	Weight gain *N* = 2,390 (%)	Weight loss *N* = 1,031 (%)	*p*-value
Gender				< 0.001
Female	2,133 (64.3)	1,588 (66.4)	804 (78.0)	
Male	1,183 (35.7)	802 (33.6)	227 (22.0)	
Age group (years)				0.004
18–24	198 (6.0)	113 (4.7)	68 (6.6)	
25–49	2,860 (86.2)	2046 (85.6)	894 (86.4)	
≥ 50	258 (7.8)	231 (9.7)	69 (6.7)	
Education				< 0.001
None/Primary	600 (18.2)	579 (24.4)	232 (22.6)	
Secondary	1,437 (43.6)	1,043 (43.9)	438 (42.6)	
Tertiary	1,257 (38.2)	755 (31.8)	357 (34.8)	
Occupation				0.004
Unemployed/Retired	528 (16.0)	382 (16.0)	208 (20.2)	
Employed	2,773 (84.0)	2000 (84.0)	821 (79.8)	
Marital status				< 0.001
Single	973 (29.3)	705 (29.5)	228 (22.1)	
Married	1907 (57.5)	1,272 (53.2)	667 (64.7)	
Separated	178 (5.4)	163 (6.8)	58 (5.6)	
Widowed	258 (7.8)	250 (10.5)	78 (7.6)	
Year of enrollment				0.002
2010	633 (19.1)	533 (22.3)	200 (19.4)	
2011	653 (19.7)	492 (20.6)	174 (16.9)	
2012	485 (14.6)	327 (13.7)	182 (17.7)	
2013	683 (20.6)	446 (18.7)	232 (22.5)	
2014	265 (8.0)	205 (8.6)	77 (7.5)	
2015	243 (7.3)	160 (6.7)	69 (6.7)	
2016	153 (4.6)	75 (3.1)	38 (3.7)	
2017	77 (2.3)	60 (2.5)	25 (2.4)	
2018	81 (2.4)	61 (2.6)	17 (1.6)	
2019	43 (1.3)	31 (1.3)	17 (1.6)	
Co-morbidities present				
Anaemia	790 (23.9)	896 (37.6)	332 (32.2)	< 0.001
Tuberculosis	87 (2.6)	216 (9.0)	36 (3.5)	< 0.001
Hypertension	709 (21.4)	412 (17.3)	206 (20.0)	0.001
Diabetes	48 (1.5)	21 (0.9)	14 (1.4)	0.143
Hepatitis B	149 (5.3)	136 (6.6)	45 (5.1)	0.101
Hepatitis C	58 (2.1)	63 (3.1)	19 (2.2)	0.073
WHO staging, at enrollment				< 0.001
Stage 1 and 2	1729 (64.0)	977 (46.8)	484 (60.6)	
Stage 3 and 4	971 (36.0)	1,110 (53.2)	315 (39.4)	
CD4 counts, cells/μL				< 0.001
≤ 200	1,172 (35.4)	1,299 (54.4)	395 (38.4)	
≥ 201	2,136 (64.6)	1,088 (45.6)	634 (61.6)	
Viral load, copies/ml				< 0.001
≤ 100,000	2,178 (71.4)	1,325 (59.9)	690 (74.1)	
≥ 100,001	871 (28.6)	888 (40.1)	241 (25.9)	
On ART regimen (at 6 months visit)				< 0.001
No	481 (14.5)	206 (8.6)	206 (20.0)	
Yes	2,835 (85.5)	2,184 (91.4)	825 (80.0)	

WHO, World Health Organization; CD4, Cluster of Differentiation 4; ART, Antiretroviral therapy, Neutral weight change – (± 5%), Weight gain – > (+5%), Weight loss - > (−5%). The participant’s characteristics are at baseline clinic visit.

**Table 5:** Predictors of absolute weight gain (in kg) using univariable and multivariable logistic regression.

Characteristics	Weight gain			
	Unadjusted odds ratio (95% CI)	*p*-value	Adjusted odds ratio (95% CI)	*p*-value
Age groups, years		0.006		0.515
18–24	1.00		1.00	
25–49	1.20 (0.95–1.52)		1.06 (0.77–1.46)	
≥ 50	1.55 (1.16–2.06)		1.21 (0.82–1.81)	
Gender		< 0.001		< 0.001
Female	1.00		1.00	
Male	1.24 (1.11–1.38)		1.35 (1.16–1.56)	
Education		0.002		0.084
None/Primary	1.00		1.00	
Secondary	0.85 (0.74–0.97)		0.82 (0.69–0.98)	
Tertiary	0.76 (0.67–0.89)		0.86 (0.71–1.03)	
Marital status		< 0.001		0.003
Single	1.00		1.00	
Married	0.89 (0.73–1.09)		0.82 (0.70–0.96)	
Separated	0.74 (0.62–0.89)		1.24 (0.93–1.67)	
Widowed	1.10 (0.84–1.45)		1.00 (0.77–1.30)	
Occupational status		0.421		0.848
Unemployed/Retired	1.00		1.00	
Employed	1.06 (0.92–1.22)		0.98 (0.82–1.18)	
Co-morbidity, present				
Hypertension	0.82 (0.72–0.94)	0.003	0.81 (0.70–0.94)	0.006
Diabetes mellitus	0.84 (0.53–1.31)	0.437	0.82 (0.48–1.38)	0.453
Tuberculosis	2.67 (2.07–3.44)	< 0.001	2.17 (1.56–3.02)	< 0.001
Anaemia	1.37 (1.22–1.53)	< 0.001	1.21 (1.04–1.40)	0.016
Hepatitis B	1.08 (0.86–1.36)	0.516	1.00 (0.76–1.31)	1.000
Hepatitis C	1.15 (0.81–1.63)	0.444	1.16 (0.77–1.74)	0.475
WHO Stage		< 0.001		< 0.001
1–2	1.00		1.00	
3–4	1.69 (1.51–1.88)		1.33 (1.15–1.53)	
Viral load, ≥ 100,001 copies/ml	1.51 (1.34–1.69)	< 0.001	1.21 (1.04–1.40)	0.011
CD4 ≤ 200 cells/μL	1.74 (1.57–1.93)	< 0.001	1.34 (1.17–1.54)	< 0.001
ART regimen base		0.001		< 0.001
Nevirapine	1.00		1.00	
Efavirenz	0.82 (0.74–0.92)		0.69 (0.59–0.80)	
Protease inhibitors	1.29 (0.76–2.11)		0.60 (0.18–2.02)	
Dolutegravir/Others	0.71 (0.46–1.08)		0.66 (0.39–1.12)	
Year of enrollment		0.001		0.179
2010	1.00		1.00	
2011	0.91 (0.77–1.08)		0.97 (0.79–1.18)	
2012	0.76 (0.63–0.91)		0.90 (0.72–1.14)	
2013	0.79 (0.67–0.93)		0.90 (0.74–1.10)	
2014	0.94 (0.76–1.16)		1.14 (0.84–1.54)	
2015	0.75 (0.60–0.94)		0.86 (0.65–1.13)	
2016	0.56 (0.43–0.74)		0.74 (0.53–1.04)	
2017	0.89 (0.64–1.25)		1.05 (0.68–1.64)	
2018	0.86 (0.61–1.21)		1.49 (0.79–2.80)	
2019	0.69 (0.44–1.09)		3.26 (0.94–11.25)	

The participants’ characteristics are at baseline clinic visit. Multivariable regression model was adjusted for age and gender.

**Table 6:** Predictors of differential weight gain (in percentages) using univariable and multivariable logistic regression.

Characteristics	Weight gain			
	Unadjusted odds ratio (95% CI)	*p*-value	Adjusted odds ratio (95% CI)	*p*-value
Age groups, years		0.001		0.290
18–24	1.00		1.00	
25–49	1.39 (1.08–1.80)		1.28 (0.91–1.82)	
≥ 50	1.76 (1.30–2.38)		1.40 (0.92–2.13)	
Gender		0.418		0.155
Female	1.00		1.00	
Male	1.05 (0.94–1.17)		1.12 (0.96–1.31)	
Education		< 0.001		0.023
None/Primary	1.00		1.00	
Secondary	0.79 (0.68–0.90)		0.80 (0.67–0.95)	
Tertiary	0.64 (0.55–0.74)		0.79 (0.65–0.95)	
Marital status		< 0.001		0.027
Single	1.00		1.00	
Married	0.89 (0.79–1.01)		0.89 (0.76–1.05)	
Separated	1.25 (0.99–1.58)		1.25 (0.93–1.68)	
Widowed	1.35 (1.10–1.65)		1.16 (0.89–1.52)	
Occupational status		0.569		0.631
Unemployed/Retired	1.00		1.00	
Employed	1.04 (0.90–1.21)		0.95 (0.79–1.16)	
Co-morbidity, present				
Hypertension	0.76 (0.67–0.88)	< 0.001	0.77 (0.64–0.92)	0.004
Diabetes mellitus	0.58 (0.35–0.97)	0.039	0.56 (0.31–1.01)	0.054
Tuberculosis	3.49 (2.75–4.44)	< 0.001	2.50 (1.82–3.42)	< 0.001
Anaemia	1.72 (1.54–1.93)	< 0.001	1.30 (1.12–1.52)	0.001
Hepatitis B	1.21 (0.95–1.53)	0.118	1.18 (0.89–1.55)	0.257
Hepatitis C	1.42 (1.00–2.02)	0.048	1.45 (0.96–2.20)	0.077
WHO Stage		< 0.001		< 0.001
1–2	1.00		1.00	
3–4	2.01 (1.80–2.25)		1.54 (1.33–1.79)	
Viral load, ≥ 100,001 copies/ml	1.77 (1.58–1.99)	< 0.001	1.36 (1.17–1.58)	< 0.001
CD4 ≤ 200 cells/μL	1.74 (1.57–1.93)	< 0.001	1.46 (1.26–1.68)	< 0.001
ART regimen base		0.020		0.003
Nevirapine	1.00		1.00	
Efavirenz	0.89 (0.80–0.99)		0.78 (0.67–0.92)	
Protease inhibitors	1.30 (0.80–2.11)		0.35 (0.09–1.35)	
Dolutegravir/Others	0.60 (0.37–0.96)		0.51 (0.28–0.93)	
Year of enrollment		< 0.001		0.027
2010	1.00		1.00	
2011	0.86 (0.73–1.02)		0.83 (0.68–1.02)	
2012	0.72 (0.60–0.86)		0.86 (0.68–1.09)	
2013	0.72 (0.60–0.85)		0.75 (0.61–0.92)	
2014	0.84 (0.68–1.04)		0.97 (0.70–1.33)	
2015	0.71 (0.56–0.89)		0.79 (0.59–1.06)	
2016	0.56 (0.43–0.74)		0.67 (0.47–0.97)	
2017	0.80 (0.56–1.12)		1.12 (0.70–1.77)	
2018	0.83 (0.59–1.18)		1.33 (0.69–2.55)	
2019	0.76 (0.48–1.22)		3.25 (0.99–10.68)	

The participants’ characteristics are at baseline clinic visit. Multivariable regression model was adjusted for age and gender.

Furthermore, exploring the predictors of weight gain using weight differentials expressed in percentages revealed similar findings. Study participants with comorbid tuberculosis (AOR: 2.50, 95% CI: 1.82–3.42), anaemia (AOR: 1.30, 95% CI: 1.12–1.52), CD4 counts ≤ 200 cells/μL (AOR: 1.46, 95% CI: 1.26–1.68), viral load ≥ 100,001 copies/ml (AOR: 1.36, 95% CI: 1.17–1.58), and WHO Clinical stages 3 and 4 (AOR: 1.54, 95% CI: 1.33–1.79) at presentation to the ART clinic were more likely to have gained weight at their 6-month follow-up clinic visit. Conversely, study participants with comorbid hypertension (AOR: 0.77, 95% CI: 0.64–0.92), secondary (AOR: 0.80, 95% CI: 0.67–0.95) and tertiary (AOR: 0.79, 95% CI: 0.65–0.95) educational levels, and year of enrollment (2013, AOR: 0.75, 95% CI: 0.61–0.92; 2016, AOR: 0.67, 95% CI: 0.47–0.97) had less likelihood of weight gain (Table 6).

## Discussion

Our study highlights weight changes observed within six months of care offered to.

PLWH at a large ART clinic in Lagos, Nigeria. Our findings reaffirm the impact of gender, anaemia, hypertension, tuberculosis, advanced HIV disease (WHO clinical stages 3 and 4, low CD4 counts) and high viral load on weight changes in the study population.

Although male gender was associated with weight gain, similar to findings in Ethiopia and other low-resource settings (30, 31), some studies reported greater weight gain following ART initiation in females (12, 32) while others found no difference by gender (33, 34). The weight difference observed could be attributed to different ART regimen compositions, prevailing socioeconomic conditions (such as poverty level, employment status, food security), dietary patterns, and body composition of PLWH before ART initiation (30, 31, 33). The patriarchal system, economic privileges, and access to nutritious food disproportionately favor males, potentially contributing to the disparity in weight gain observed. While gender was associated with weight gain, other health conditions like hypertension had the opposite effect.

Hypertension was associated with lower odds of weight gain in this study, which may be attributed to PLWH with co-morbid hypertension adhering to lifestyle advice to eat a healthy diet and exercise regularly (35, 36). Although the association of overweight/obesity with prevalent or incident hypertension among PLWH has been reported in several studies, there is a dearth of evidence on weight changes following hypertension diagnosis in this population (37, 38). However, our finding would require validation among the HIV cohort.

Study participants with Advanced HIV disease (CD4 counts ≤ 200 cells/μL, and WHO Clinical Stage 3 and 4) and high viral load (≥ 100,000 copies/ml) at baseline clinic visit had comparatively higher weight gain in concordance with other studies (15, 39, 40). Following ART initiation, the interruption of HIV adverse effects (sustained inflammation and accelerated catabolism) and resolution of opportunistic infections, especially in the gastrointestinal tract (GIT) is thought to be responsible for the return to health and resultant weight gain (41, 42). However, other studies have shown that PLWH without advanced HIV disease at ART initiation were also associated with weight gain (43, 44). This disparity may be due to the study population composition (age, gender, and BMI distribution) as well as burden of opportunistic infections and AIDS-defining conditions. Furthermore, other studies showed no association between viral load counts at baseline and weight changes following ART initiation (45, 46).

Anaemia at baseline clinic visit was associated with weight gain at the 6th-month clinic visit, similar to findings in the USA (20). However, this contrasts with other studies that have shown impaired weight gain among PLWH with anaemia at ART initiation (47, 48). Anaemia among PLWH is mainly caused by persistent chronic inflammation, opportunistic infections as well as HIV infection (26, 49). Furthermore, anaemia has been associated with advanced HIV disease, HIV disease progression, and mortality (23, 50).

Wasting syndrome, with weight loss being a key clinical feature, is associated with both HIV and tuberculosis infections (51, 52). In addition, plasma leptin levels responsible for appetite and food intake are reduced in the presence of tuberculosis infection (53). Following initiation of appropriate therapy (HIV and tuberculosis treatment), comparatively higher weight gains have been recorded among PLWH with co-morbid tuberculosis when compared to those without tuberculosis, similar to a study in Kenya (54).

The majority of our study participants received a non-nucleoside reverse transcriptase inhibitor (NNRTI) (either nevirapine or efavirenz) at ART initiation as the preferred first-line regimen. Weight gain observed amongst PLWH on efavirenz-based regimen lagged behind those initiated on nevirapine-based regimen. Weight gain attributed to dolutegravir-based regimen could not be fully demonstrated as its use was introduced only in 2019 at our ART clinic (i.e., several years after the commencement of this study). Due to the late introduction of dolutegravir based regimen, the effects of dolutegravir did not align with findings from other settings. Furthermore, Protease inhibitors (PI) based regimens are used as second-line treatment options in the clinic. Thus, a smaller proportion of the study population were on dolutegravir and PI based regimens. The use of dolutegravir based regimen had been reported to increase weight among ART-naïve PLWH significantly (55). Although dolutegravir use was limited in this study, future studies should elucidate its association with weight gain among PLWH in Nigeria. Nevertheless, this study aligns with weight gain among PLWH irrespective of the regimen initiated (20).

Weight gain following ART initiation has been shown to be a harbinger of good treatment outcome. However, there are potentials for the development of cardiometabolic disorders such as diabetes, obesity, hypertension and dyslipidaemia (13, 56). Thus, healthcare providers should monitor the long-term impact of ARVs and perceived increased cardiovascular disease risks. Appropriate management of diet, lifestyle modifications, and regular assessment of weight (and BMI) could mitigate non-AIDS adverse events following ART initiation.

## Strengths and limitations

This study explored weight changes among ART-naïve PLWH initiating therapy over a ten-year period in Lagos, Nigeria. We believe our findings are generalisable with other HIV care settings despite being a single site study due to the large sample size and ease of access to the ART clinic. Study limitations include absence of data on dietary intake, physical activity, cotrimoxazole prophylaxis (duration and strength), and adherence to ARVs. In addition, the study did not account for co-morbid HIV/AIDS-related (infectious) opportunistic infections (aside from tuberculosis) and adverse events following ART initiation. Finally, the 6-month follow-up period may also limit the visualization of the long-term effects of ARVs on weight gain in PLWH.

## Conclusion

This study explored weight changes and associated predictors among ART-naïve PLWH initiating therapy in Lagos, Nigeria. Weight gain was associated with male gender, advanced HIV disease (low CD4 counts and WHO Clinical stages 3 and 4), high viral load, anaemia, and comorbid tuberculosis at presentation to the ART clinic. Excess weight gain following ART initiation has emerged as a public health concern with particular emphasis on the associated clinical sequelae (nutritional and metabolic disorders) in the face of scarce resources in the health ecosphere within the country. Beyond favorable treatment outcomes (virological suppression, weight gain, absence of AIDS-defining illness), the monitoring of increased cardiometabolic disease risk should be incorporated into the HIV care continuum to abate the growing threat of NCDs in this cohort.

## Data Availability

The original contributions presented in the study are included in the article/Supplementary material, further inquiries can be directed to the corresponding author.
