# Supplementary material for: Weight changes among antiretroviral therapy-naïve people living with human immunodeficiency virus in Lagos, Nigeria
Source: Front Public Health. 2025 May 13;13:1545676. doi: 10.3389/fpubh.2025.1545676 (PMC12106384; doi:10.3389/fpubh.2025.1545676)
Supplement: Supplementary file 1 [file Table_1.docx]

**SUPPLEMENTARY MATERIALS**

Supplementary Table 1: Coefficients for multicollinearity

| Variables | Collinearity Tolerance | Statistics VIF |
| --- | --- | --- |
| Gender | 0.845 | 1.183 |
| Age group | 0.857 | 1.166 |
| Education | 0.918 | 1.090 |
| Marital Status | 0.859 | 1.164 |
| Occupation | 0.939 | 1.065 |
| Hypertension | 0.946 | 1.057 |
| Diabetes Mellitus | 0.987 | 1.013 |
| Tuberculosis | 0.917 | 1.091 |
| Anaemia | 0.859 | 1.164 |
| Hepatitis B | 0.985 | 1.015 |
| Hepatitis C | 0.992 | 1.008 |
| WHO clinical staging | 0.822 | 1.217 |
| Viral load | 0.871 | 1.148 |
| CD4 counts | 0.839 | 1.192 |
| ART regimen | 0.825 | 1.212 |
| Year of enrollment | 0.844 | 1.185 |

Supplementary Table 2: Association between Antiretroviral therapy base regimen and absolute weight changes (in kg)

| Weight changes  | Antiretroviral therapy (ART) regimen base | | | |  |
| --- | --- | --- | --- | --- | --- |
|  | Nevirapine  N (%) | Efavirenz  N (%) | Protease Inhibitors  N (%) | Dolutegravir and others N (%) | p-value |
| Neutral | 503 (34.6) | 905 (62.3) | 19 (1.3) | 25 (1.7) | 0.487 |
| Gain | 1144 (38.3) | 1762 (59.0) | 41 (1.4) | 41 (1.4) | 0.001 |
| Loss | 466 (33.2) | 906 (64.5) | 8 (0.6) | 24 (1.7) | 0.003 |
